# A Patient with Advanced Gastric Cancer Presenting with Extremely Large Uterine Fibroid Tumor

**DOI:** 10.1155/2014/760913

**Published:** 2014-02-12

**Authors:** Kwang-Kuk Park, Song-I Yang

**Affiliations:** Department of Surgery, Kosin University College of Medicine, 34 Amnam-dong, Seo-gu, Busan 602-703, Republic of Korea

## Abstract

*Introduction*. Uterine fibroid tumors (uterine leiomyomas) are the most common benign uterine tumors. The incidence of uterine fibroid tumors increases in older women and may occur in more than 30% of women aged 40 to 60. Many uterine fibroid tumors are asymptomatic and are diagnosed incidentally. *Case Presentation*. A 44-year-old woman was admitted to our hospital with general weakness, dyspepsia, abdominal distension, and a palpable abdominal mass. An abdominal computed tomography scan showed a huge tumor mass in the abdomen which was compressing the intestine and urinary bladder. Gastroduodenal endoscopic and biopsy results showed a Borrmann type IV gastric adenocarcinoma. The patient was diagnosed with gastric cancer with disseminated peritoneal carcinomatosis. She underwent a hysterectomy with both salphingo-oophorectomy and bypass gastrojejunostomy. Simultaneous uterine fibroid tumor with other malignancies is generally observed without resection. But in this case, a surgical resection was required to resolve an intestinal obstruction and to exclude the possibility of a metastatic tumor. *Conclusion*. When a large pelvic or ovarian mass is detected in gastrointestinal malignancy patients, physicians try to exclude the presence of a Krukenberg tumor. If the tumors cause certain symptoms, surgical resection is recommended to resolve symptoms and to exclude a metastatic tumor.

## 1. Introduction

Gastric cancer is one of the most commonly diagnosed malignancies in South Korea. Female patients with advanced gastric cancer, in particular premenopausal patients, are also often found to have Krukenberg tumors. Because of this, when a large pelvic or ovarian mass is detected in gastrointestinal malignancy patients, physicians try to exclude the presence of a Krukenberg tumor. The incidence of uterine fibroid tumors increases as women grow older and these tumors may occur from 4 percent in women 20 to 30 years of age to 11 to 18 percent in women 30 to 40 years of age and 33 percent in women 40 to 60 years [1]. Many tumors are asymptomatic and are diagnosed incidentally. Although a causal relationship has not been established, uterine fibroid tumors are associated with menorrhagia, pelvic pain, pelvic or urinary obstructive symptoms, infertility, and pregnancy loss. A patient recently visited our hospital with sudden onset abdominal distension and indigestion. The patient was diagnosed with stomach cancer and suspected metastatic uterine tumors. We performed a hysterectomy with both a salphingo-oophorectomy and a bypass gastrojejunostomy. A very large uterine mass was histologically revealed to be a uterine fibroid tumor, not a Krukenberg tumor. We report this first case of an extremely large uterine fibroid tumor in a patient with advanced gastric cancer.

## 2. Case Presentation

**Figure 1:**
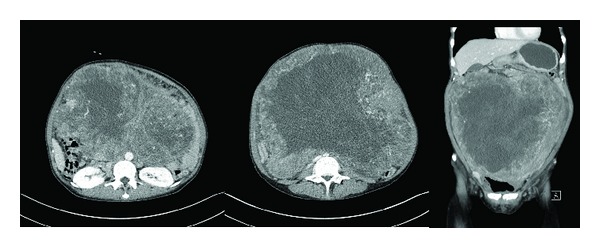
Abdominal computed tomography (CT) showing a huge solid mass occupying whole pelvis and abdomen.

**Figure 2:**
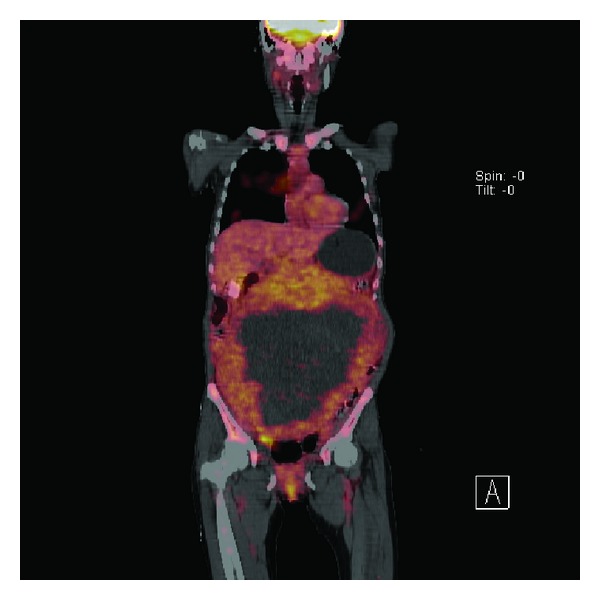
F-18 fludeoxyglucose (FDG) positron emission tomography/computed tomography (PET/CT) images.

**Figure 3:**
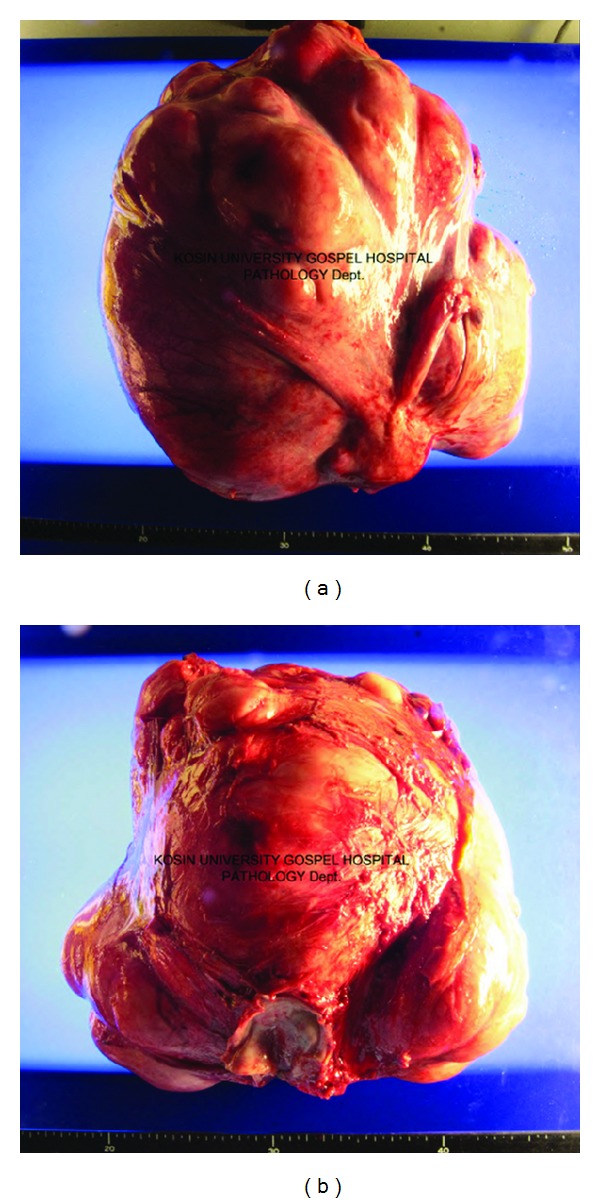
Macroscopic view of uterine fibroid tumor.

A 44-year-old woman was admitted to our hospital with general weakness, dyspepsia, abdominal distension, and a palpable mass that had been present for two weeks. The patient appeared pale and was chronically ill. She stated that she had lost 6 kg over the last two months. On physical examination, she was oriented to time, place, and person. Her vital signs were as follows: blood pressure of 130/75 mmHg, pulse rate of 83 beats/min, and respiration rate of 24 breaths/min. Abdominal examination revealed a mass that was palpable over the entire abdomen. There was no jaundice, cyanosis, or diaphoresis. Neurological and cardiac examinations did not exhibit any pathological findings. Tumor marker levels including carcinoembryonic antigen (CEA), human chorion gonadotropin (HCG), CA19-9, and CA15-3 were all within normal range, but CA125 was high at 171 U/mL. An abdominal computed tomography scan (Figure 1) showed a huge tumor mass in the abdomen which was compressing the intestine and urinary bladder. Gastroduodenal endoscopic and biopsy results showed a Borrmann type IV gastric adenocarcinoma in the prepyloric antrum with gastric outlet obstruction. A F-18 fluorodeoxyglucose (FDG) positron emission tomography/computed tomography (PET-CT) showed a huge pelvic mass and increased FDG uptake from the main pelvic mass and multiple hypermetabolism in the mesentery and peritoneum (Figure 2). An exploratory laparotomy was performed with a long midline incision. After the peritoneal cavity was opened, an enormous circumscribed mass measuring 28.0 × 20.0 × 27.0 cm (Figure 3) was found to be displacing the bowel to the abdominal adhesions periphery. The mass originated from the right corner of the uterus. Multiple peritoneal nodules resembling peritoneal carcinomatosis were observed and a frozen biopsy was carried out. She was diagnosed with peritoneal carcinomatosis of gastric cancer. A total hysterectomy with both salphingo-oophorectomy and bypass gastrojejunostomy was performed. The tumor was sent for histological and cytological assessment, which did not reveal any evidence of malignancy. Due to maladaptation of lung capacity, she complained of difficulty breathing. On the third postoperative day, her breathing was comfortable. The postoperative course was uneventful, and the patient had no further complications. The patient is currently receiving chemotherapy with S-1 plus CDDP (cisplatin), according to the following regimen: S-1 (50 mg/m (2) p.o. b.i.d. from D1 to 14) and cisplatin (70 mg/m (2) on D1), repeated every 3 weeks.

## 3. Discussion

Female patients with advanced gastric cancer, particularly in the premenopausal state, are subject to Krukenberg tumors [2]. Uterine fibroid tumors are the most common female reproductive tract tumors. They are usually of a unicellular origin, and their growth rate is influenced by estrogen, growth hormone, and progesterone. Although exact process of tumor formation has not been elucidated, uterine fibroid tumors arise during reproductive years and tend to enlarge during pregnancy and regress after menopause.

The use of estrogen agonists is associated with an increased incidence of fibroid tumors [3], and growth hormone appears to act synergistically with estradiol in affecting the growth of uterine fibroid tumors. Some studies have shown increased estrogen receptor mRNA in fibroids compared with normal myometrium [4]. Fibroids overexpress aromatase p450, a synthetase which produces estrogen from androgens, suggesting that local estrogen may play a role in the growth of uterine fibroids [5]. Sex-steroid action is mediated partially via other growth factors such as epidermal growth factor and insulin-like growth factor [6]. Estrogen upregulates epidermal growth factors and transforming growth factor-beta1 and transforming growth factor-beta3, all of which play a role in the growth of uterine fibroids [7]. Progesterone is thought to exert a dual action, as it can promote fibroid growth but also may have an inhibitory effect on fibroid growth through downregulating insulin-like growth factor-1 (IGF-1) expression [6]. Several other studies have reported an increased incidence of uterine fibroid tumors in black women [8].

Pelvic pain and pressure are less commonly attributed to uterine fibroid tumors. Individual case reports have described very large tumors that result in pelvic discomfort, respiratory failure, urinary symptoms, and constipation [9–11]. It has been reported that solitary or multiple tumors are possible and may rarely present in a botryose shape [12]. Tumor sizes have been reported to range from microscopic to 3400 g [13]. The fibroid tumor reported here was 7990 g.

The role of uterine fibroid tumors in infertility is controversial. Many studies examining the relationship between these tumors and infertility are retrospective and nonrandomized. Current evidence suggests that submucosal and intramural fibroid tumors that distort the uterine cavity can impair in vitro fertilization attempts [14].

Uterine myomas are classified into subgroups according to their position and relationship to the uterine layers. These tumors become symptomatic based on their position within the uterus and their size. Tumors are usually distinguished by the following characteristics: (a) intramural myomas; (b) submucosal (endocavitary) myomas, which can be pedunculated or sessile and can extend into the myometrium; (c) subserosal myomas, which can be pedunculated or sessile and are located just beneath the covering peritoneum of the uterine corpus; (d) isthmus or cervical myomas; and (e) extrauterine (intraligamentary or intraovarian) myomas [15].

The treatment for uterine fibroid tumors with no symptoms and a small size is observation at intervals of 6 months. In terms of medical therapy, GnRH agonists, medroxyprogesterone acetate, danazol, and mifepristone (RU 486), which reduces the serum progesterone and estrogen, were reported to reduce the fibroid volume. Surgical treatment can be considered in cases of abnormal bleeding with sustained endometrial hyperplasia or when no improvement is seen with palliative therapy, and uterine artery embolization has been used recently in these cases. A total hysterectomy or myomectomy should be considered based on the patient's age, parity, and future pregnancy plans. Decreases in serum estrogen levels are expected after menopause, which may cause a decrease in the size of the myoma, so surgical removal is therefore not required in most patients who are approaching menopause [13].

## 4. Conclusion

In this case, a surgical resection was required to resolve an intestinal obstruction and to exclude the possibility of a Krukenberg tumor. We report a surgical resected uterine fibroid tumor in a patient with advanced gastric cancer.
